# Causal assessment of the relationship between diet, lifestyle, and stroke: A two-sample Mendelian randomization study

**DOI:** 10.1097/MD.0000000000042563

**Published:** 2025-05-30

**Authors:** Mohammad A. Jareebi

**Affiliations:** a Family and Community Medicine Department, Faculty of Medicine, Jazan University, Jazan City, Jazan, Saudi Arabia.

**Keywords:** diet, lifestyle, MR, stroke

## Abstract

Stroke, a debilitating neurological disorder with significant global morbidity and mortality, is the focus of this study. Observational studies are susceptible to confounding and limited generalizability. To mitigate confounding and reverse causation, this study employs Mendelian randomization to establish a causal relationship between modifiable risk factors and stroke. Data from the UK Biobank (n = 461,880) and FinnGen (n = 180,862) cohorts were analyzed, using genetic variants as instrumental variables. The study investigates relationships between stroke risk and genetically predicted exposures such as salad and fruit consumption, cheese intake, coffee, body mass index, maternal smoking, and smoking behavior. A genetically estimated higher intake of salads and fruits was observed to be associated with a decreased risk of stroke in both FinnGen and the UK Biobank (salads: odds ratio [OR] 0.98, 95% confidence interval [CI]: 0.97–0.99, *P* = .006; fruits: OR 0.98, 95% CI: 0.97–0.99, *P* = .019). Conversely, both maternal smoking (OR 1.003, 95% CI: 1.001–1.01, *P* = .006) and smoking behavior (OR 1.04, 95% CI: 1.02–1.06, *P* < .001) were associated with an increased risk of stroke. Physical activity demonstrated a protective effect (OR 0.994, 95% CI: 0.97–0.998, *P* = .05). In the FinnGen cohort, similar but more pronounced protective effects were observed for fruit consumption (OR 0.60, 95% CI: 0.45–0.82, *P* = .04) and physical activity (OR 0.78, 95% CI: 0.62–0.98, *P* < .05). Additionally, maternal smoking in this population was associated with a substantially increased stroke risk (OR 2.20, 95% CI: 1.27–3.01, *P* < .001). This study underscores the preventive roles of a healthy diet, smoking cessation, and adopting a healthy lifestyle in mitigating the risks of stroke. Further research is warranted to delve into the mechanisms underlying these risks and protective factors.

## 1. Introduction

According to the World Health Organization (WHO), stroke is a catastrophic cerebrovascular disorder that has become the second leading cause of mortality, affecting millions of people worldwide. In 2019, there were approximately 12.2 million new stroke cases worldwide, with 101 million people living with its consequences. Modifiable risk factors are the focus of evidence-based preventative measures, crucial for reducing the burden on public health.^[1]^

As stroke is a major manifestation of cardiovascular disease (CVD), its prevention is crucial for reducing the global burden of CVD-related morbidity and mortality. Early onset of some cardiovascular risk factors predicts the onset of other risk factors in the long term. Addressing modifiable cardiometabolic risk factors, such as hypertension, obesity, dyslipidemia, and diabetes, at an early stage is fundamental to comprehensive CVD and stroke-prevention strategies.^[2]^

Stroke is a complex disease influenced by both genetic and environmental factors. Major contributors include diet and lifestyle, impacting weight management and blood pressure, which extend beyond directly lowering stroke risk.^[3,4]^ However, disentangling their causal roles from observational confounding effects remains challenging. Traditional epidemiological studies, while informative, can be hindered by confounding biases, unmeasured variables, and reverse causality. The intricate interplay between diet, lifestyle, and stroke risk warrants thorough investigation.^[5]^

Dietary patterns that encompass specific food groups and nutrients play a significant role in influencing stroke incidence.^[6]^ An increased intake of fruits, vegetables, and unsaturated fats, along with a reduction in red meat and processed foods, has been associated with a lower stroke risk.^[7,8]^ However, establishing their causal roles has been elusive. Similarly, lifestyle factors such as physical activity, smoking, and alcohol consumption exert complex influences on stroke risk.^[5]^ While observational studies suggest their association with stroke, establishing causation remains crucial for effective preventive strategies.

Mendelian randomization (MR) emerges as a powerful tool offering robust insights into potential causal relationships in this context.^[9]^ MR leverages genetic variants, randomly inherited and uninfluenced by confounding factors, as instrumental variable to mimic the exposure (diet/lifestyle) and estimate its unbiased causal effect on the outcome (stroke risk). This robust approach overcomes limitations of observational studies, providing more reliable evidence for causal influence.^[9]^

In contrast to earlier single-cohort studies prone to confounding and limited generalizability, this two-sample MR study controls 2 large, diverse cohorts (UK Biobank [UKB] and FinnGen) to simultaneously evaluate a wide range of diet and lifestyle factors influencing stroke risk. This multi-cohort approach not only mitigates potential confounding by diversifying the study population but also enhances the generalizability of our findings. By investigating causal relationships across disparate populations, we ensure our conclusions are relevant to a wider population, laying a stronger foundation for informing public health recommendations. This study aimed to provide robust evidence for causal relationships between diet, lifestyle, and stroke risk, informing targeted interventions and preventive strategies.

## 2. Materials and methods

### 2.1. Study type

**Figure 1.**
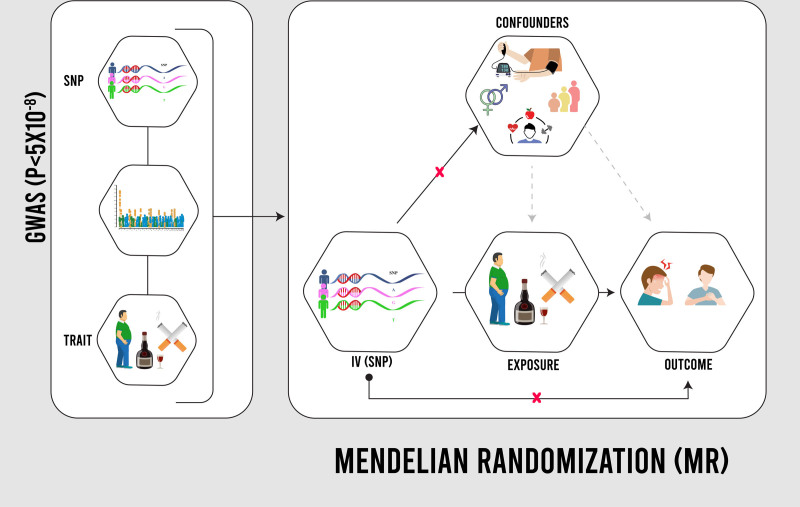
Mendelian randomization design.

This study is a genetic analysis utilizing summary-level data from two-sample MR. MR is a statistical method that utilizes genetic variations, specifically single nucleotide polymorphisms (SNPs), to determine causal relationships between modifiable risk factors and health outcomes. By leveraging genome-wide association studies, MR minimizes confounding biases and reverse causation inherent in observational research. This approach relies on key assumptions: SNPs must strongly associate with the risk factor, not directly influence the outcome, and remain independent of confounders. This study employed a two-sample MR approach, sourcing exposure and outcome data from different populations of the same ancestry, ensuring robust analysis of causal relationships between risk factors and migraine (Fig. 1). This two-sample MR analysis followed the STROBE guidelines for reporting observational studies.

In MR analysis, the acquisition of exposure and outcome data from the same population is termed one-sample, while sourcing data from disparate populations is denoted as two-sample. Regardless of whether the analysis is one-sample or two-sample, the data should emanate from the same ancestral background, such as European ancestry.^[10]^

### 2.2. Study population (the UKB and FinnGen cohorts)

The UKB comprises a substantial prospective cohort, encompassing approximately 502,000 individuals who underwent assessment at one of the 22 evaluation centers situated across England, Scotland, and Wales between 2006 and 2010. The examination protocol encompassed diverse medical, psychosocial, and anthropometric parameters, alongside self-reported and doctor-diagnosed medical conditions.^[11]^ FinnGen represents a genetic research initiative established in Finland with the primary goal of amassing genetic data from 500,000 Finnish participants. Its mission revolves around investigating the associations between genetic factors and various diseases. Commencing in 2017, this undertaking is anticipated to conclude by 2025, with over 200,000 Finnish individuals already contributing their genetic data to support this comprehensive investigation.^[12]^ This study utilized publicly accessible summary-level data derived from these consolidated datasets.

### 2.3. Objective

The present study explored the potential causal role of diet and lifestyle in the development of stroke. Furthermore, the study investigated various potential risk factors that could influence the occurrence of stroke. These factors encompassed the initiation and intensity of smoking, historical maternal smoking, salad intake, body mass index (BMI), fruit consumption, and self-reported levels of physical activity.

### 2.4. SNPs selection

We identified relevant variables associated with diet, lifestyle, smoking, and anthropometric factors by leveraging genome-wide significant SNPs from the following consortia, including Pirastu et al,^[13]^ the UKB,^[14]^ genome-wide association studies and sequencing consortium of alcohol and nicotine use,^[15]^ Genetic Investigation of Anthropometric Traits.^[16]^ These SNPs represent genetic variations associated with specific traits, as determined through genome-wide association studies at a significance threshold of *P* < 5 × 10^‐8^.^[4]^

The study utilized a distinct set of SNPs that are used widely in the literature,^[17,18]^ for each exposure. Specifically, there were 41 SNPs for fruits intake,^[13]^ 22 SNPs for salad intake,^[14]^ 65 SNPs for cheese intake,^[14]^ 3 SNPs for coffee consumption,^[14]^ 93 SNPS for smoking initiation,^[15]^ 23 SNPs for smoking intensity,^[15]^ 16 SNPs for maternal smoking,^[19]^ 79 SNPs for BMI,^[16]^ and 11 SNPs for physical activity.^[16]^

### 2.5. Statistical analysis and genetic data integration

The principal objective of this investigation was to assess the causal relationship between diet and physical activity and stroke, a binary outcome. Genetic data related to stroke were obtained from 2 sources: UKB and FinnGen.^[11,12]^ After harmonizing the data (i.e., the process of aligning and standardizing the reporting of genetic associations to ensure that the effect of alleles for exposure and outcome is consistently expressed per additional copy of the same allele), we examined a set of SNPs for each exposure factor in relation to CAD.

MR and sensitivity analyses were conducted using the two-sample MR package in R software (version 4.2.3). The analysis involved collecting genetic data for the exposures and their corresponding outcomes. Separate MR analyses were performed for CAD using data from both the UKB and FinnGen consortia. A significance level of *P* < .05 was applied to all MR analyses, with a primary focus on the inverse variance weighted method. Additionally, we employed more restrictive MR measures, including MR-Egger, which considers increased pleiotropy to identify any potential deviations from inverse variance weighted results.^[3]^

## 3. Results

### 3.1. Genetic profiling of risk factors

A total of 353 SNPs were scrutinized concerning diverse risk factors whereby each factor underwent assessment through a spectrum of 3 to 93 SNP variants. These genetic markers were sourced from distinct consortia, each characterized by disparate sample sizes ranging from 64,949 to 632,802 individuals per risk factor (Table 1).

**Table 1:** Genetic risk factors in brief: a summary.

Exposure	No. of SNPs	Sample size	Population/consortium
Fruits	41	409,125	Pirastu et al^[14]^
Salad	22	462,933	UKB^[15]^
Cheese	65	451,486	UKB^[15]^
Coffee	3	64,949	UKB^[15]^
Smoking initiation	93	632,802	GSCAN^[16]^
Smoking intensity	23	249,752	GSCAN^[16]^
Maternal smoking	16	397,732	MRC-IEU^[20]^
Body mass index (BMI)	79	339,152	GIANT^[17]^
Number of days/weeks of vigorous physical activity	11	440,512	UKB^[15]^

GIANT = genetic investigation of anthropometric traits, GSCAN = sequencing consortium of alcohol and nicotine use, MRC-IEU = Medical Research Council (MRC) Integrative Epidemiology Unit at the University of Bristol; SNPs = single nucleotide polymorphisms, UKB = UK Biobank.

### 3.2. Stroke genetic characteristics

The risk of stroke was investigated in 2 distinct population cohorts: the UKB and the FinnGen cohort. In the UKB cohort, where strokes were diagnosed by a medical professional, a total of 461,880 individuals were included. Among them, 7055 cases were identified with doctor-diagnosed stroke, accompanied by 454,825 controls. Analogously, within the FinnGen cohort consisting of 180,862 individuals, 18,661 cases were diagnosed with stroke by a doctor, while 162,201 individuals were employed as controls in the analysis.

### 3.3. Stroke risk in the UKB cohort

The UKB consortium conducted a comprehensive investigation into genetically estimated risk factors for stroke. Noteworthy findings included a significant association between higher fruit and salad intake and a lower risk of stroke, indicated by odds ratio (OR) of 0.98 (95% confidence interval [CI]: 0.97–0.99, *P* = .019) and 0.98 (95% CI: 0.97–0.99, *P* = .006), respectively. Cheese intake also demonstrated a decreased risk of stroke (OR = 0.99, 95% CI: 0.990–0.999, *P* = .03). Conversely, coffee consumption exhibited a nonsignificant association with stroke risk (OR = 1.01, 95% CI: 0.99–1.03, *P* = .148).

The impact of smoking on stroke risk was explored, revealing significant associations for both initiation (OR = 1.003, 95% CI: 1.001–1.01, *P* = .006) and intensity (OR = 1.002, 95% CI: 0.99–1.003, *P* = .073). Maternal smoking emerged as a particularly potent risk factor, with an odds ratio of 1.04 (95% CI: 1.02–1.06, *P* < .001). In contrast, BMI displayed a modest association with stroke risk (OR = 1.001, 95% CI: 0.99–1.003, *P* = .298).

**Figure 2.**
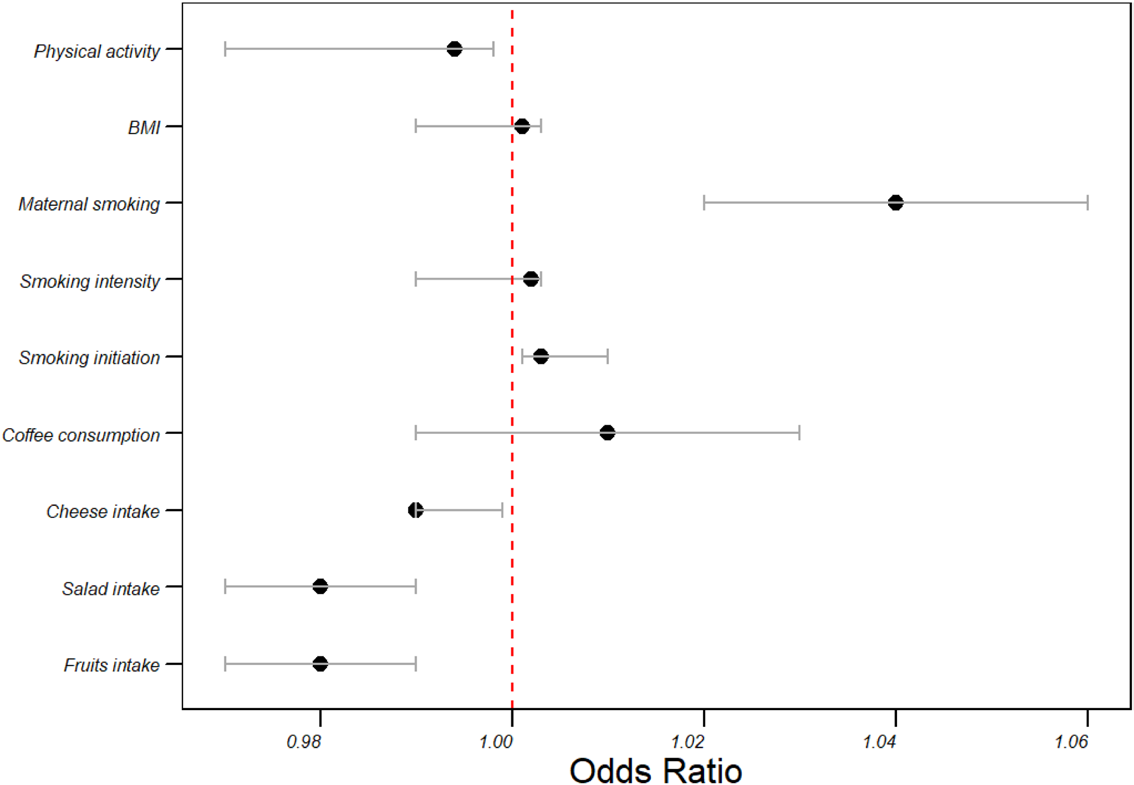
Impact of dietary patterns and lifestyle on stroke risk in the UKB cohort. UKB = UK Biobank.

Lastly, physical activity exhibited a protective effect against stroke, with an odds ratio of 0.994 (95% CI: 0.97–0.998, *P* = .05). These significant findings contribute valuable insights into the interplay of various factors in stroke risk within the UKB consortium study (Table 2 and Fig. 2).

**Table 2:** Overview of stroke findings in the UKB cohort.

Risk factor	OR (95% CI)	*P* value
Fruits intake	0.98 (0.97–0.99)	**.019**
Salad intake	0.98 (0.97–0.99)	**.006**
Cheese intake	0.99 (0.990–0.999)	**.03**
Coffee consumption	1.01 (0.99–1.03)	.148
Smoking initiation	1.003 (1.001–1.01)	**.006**
Smoking intensity	1.002 (0.99–1.003)	.073
Maternal smoking	1.04 (1.02–1.06)	**<.001**
BMI	1.001 (0.99–1.003)	.298
Physical activity	0.994 (0.97–0.998)	**.05**

CI = confidence interval, UKB = UK Biobank.

### 3.4. Stroke risk in the FinnGen cohort

In the FinnGen consortium’s analysis of genetically estimated risk factors for stroke, significant findings include the substantial protective effect of higher fruit intake, with an odds ratio of 0.60 (95% CI: 0.45–0.82, *P* = .04). Salad intake, while showing a decreased odds ratio of 0.74 (95% CI: 0.49–1.14), did not reach statistical significance (*P* = .414). Similarly, cheese intake demonstrated a trend towards lower stroke risk (OR = 0.84, 95% CI: 0.68–1.03, *P* = .129). Conversely, coffee consumption did not reveal a significant association with stroke risk (OR = 1.20, 95% CI: 0.73–1.65, *P* = .683).

**Figure 3.**
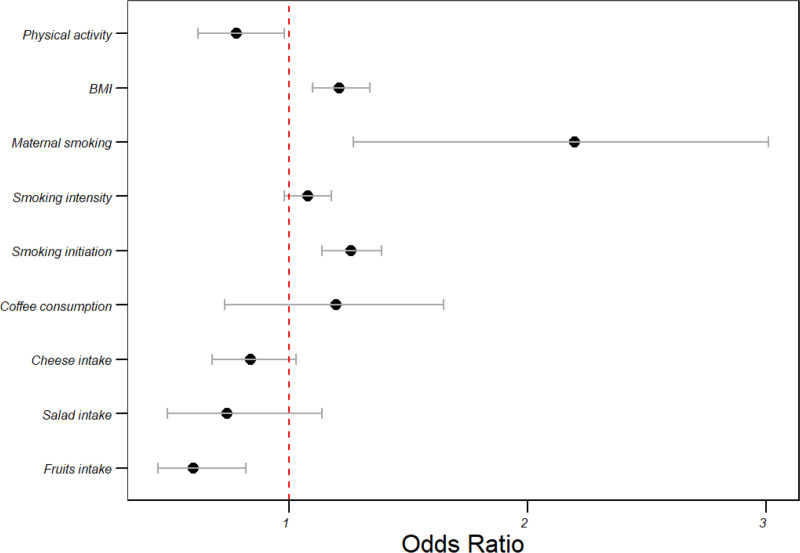
Impact of dietary patterns and lifestyle on stroke risk in the FinnGen cohort.

The impact of smoking, both in terms of initiation and intensity, was notably pronounced in this cohort with significant associations observed (smoking initiation: OR = 1.26, 95% CI: 1.14–1.39, *P* < .001; smoking intensity: OR = 1.08, 95% CI: 0.98–1.18, *P* = .126). Maternal smoking emerged as a potent risk factor for stroke in the FinnGen cohort, with a substantial odds ratio of 2.20 (95% CI: 1.27–3.01, *P* < .001). Additionally, BMI exhibited a significant positive association with stroke risk (OR = 1.21, 95% CI: 1.10–1.34, *P* = .001). On the other hand, higher physical activity levels demonstrated a protective effect against stroke, as evidenced by an odds ratio of 0.78 (95% CI: 0.62–0.98, *P* < .05). These findings contribute valuable insights into the genetic risk factors associated with stroke within the FinnGen cohort (Table 3 and Fig. 3).

**Table 3:** Overview of stroke findings in the FinnGen cohort.

Risk factor	OR (95% CI)	*P* value
Fruits intake	0.60 (0.45–0.82)	**.04**
Salad intake	0.74 (0.49–1.14)	.414
Cheese intake	0.84 (0.68–1.03)	.129
Coffee consumption	1.20 (0.73–1.65)	.683
Smoking initiation	1.26 (1.14–1.39)	**<.001**
Smoking intensity	1.08 (0.98–1.18)	.126
Maternal smoking	2.20 (1.27–3.01)	**<.001**
Body mass index (BMI)	1.21 (1.10–1.34)	**.001**
Physical activity	0.78 (0.62–0.98)	**<.05**

CI = confidence interval.

## 4. Discussion

This comprehensive two-sample MR study presented evidence of potential causal relationships between diet, lifestyle factors, and stroke risk. Using genetic variants as instrumental variables helped mitigate confounding biases common in observational studies, establishing robust causal inferences between modifiable risk factors and stroke risk. Leveraging data from 2 sizable population cohorts, FinnGen (n = 180,862) and UKB (n = 461,880), we utilized a distinct set of 353 SNPs widely recognized in the literature for each exposure.^[13–19,21,22]^

Results from both FinnGen and UKB cohorts revealed intriguing associations between genetically predicted lifestyle factors and stroke risk. Fruits and salads emerged as potential natural stroke-prevention factors, showing a significant inverse relationship between genetically predicted fruit intake and stroke risk in both populations. Similarly, salad exhibited a positive trend towards protection in both cohorts. Flavonoids (flavonols, flavones, and isoflavones) in fruits and vegetables have robust antioxidant, anti-inflammatory, and antithrombotic effects; a higher flavonol intake is inversely linked to nonfatal and fatal stroke. Similarly, carotenoids, like lycopene, known for their antioxidant properties, are associated with reduced cardiovascular disease risk.^[21]^ While not statistically significant in FinnGen, these findings align with an earlier study by Joshipura et al,^[22]^ encouraging further investigation into the specific bioactive elements in fruits and vegetables supporting their protective qualities.

The consumption of cheese presented a complex picture, with UKB suggesting a potential protective effect, while FinnGen did not find statistical significance. Conversely, in both cohorts coffee did not exhibit a statistically significant association with stroke, calling for additional research on the true effects of cheese varieties and coffee consumption on stroke risk. A positive association between BMI and stroke risk was observed in both cohorts, emphasizing the importance of maintaining a healthy weight through a balanced diet and physical activity.^[20,23]^

Notably, our findings in both cohorts revealed a strong positive association between genetically predicted smoking and stroke risk, consistent with previous studies.^[24,25]^ These studies also demonstrated a dose-dependent association, where greater genetic risk scores for smoking translated to an increased stroke risk. In the FinnGen cohort, maternal smoking history also showed a significant relationship with an increased risk of stroke, highlighting the long-term effects of maternal smoking on vascular health. This underscores the potential for lifestyle modifications, such as quitting smoking, to lower the risk of stroke, irrespective of genetic predisposition.^[26]^

The study reaffirms that leading a healthy lifestyle lowers the risk of stroke.^[27,28]^ The benefits of maintaining a healthy lifestyle were consistent across all genetic risk strata, with genetically predicted levels of physical activity consistently demonstrating a protective impact against stroke risk. In both cohorts, we found that genetically predicted levels of physical activity had a consistent protective impact against the risk of stroke. This result is consistent with previous research showing the significant benefits of including regular physical activity in one’s lifestyle to support cardiovascular health and lower stroke risk.^[29]^ This emphasizes the crucial role of regular exercise in supporting cardiovascular health and lowering stroke risk, regardless of genetic inclination.

In addition to the modifiable risk factors examined in this study, emerging evidence underscores the clinical relevance of demographic, anthropometric, and echocardiographic variables in shaping individualized stroke risk profiles. While these factors were beyond the scope of the current investigation, their potential contributions warrant consideration based on established literature. Of particular interest are anthropometric features such as mitral valve prolapse (MVP) and concave-shaped chest walls, which may confer protective effects against cardiovascular and cerebrovascular events. MVP, the most common cardiac valvular anomaly, is characterized by thickened, elongated leaflets, significant mitral regurgitation (MR), and left atrial or ventricular enlargement. Despite its structural abnormalities, MVP is associated with a generally favorable prognosis. Notably, the presence of hemodynamically significant MR in MVP patients has been hypothesized to reduce stroke risk through a “washing effect,” wherein turbulent blood flow in the left atrium mitigates blood stasis and subsequent thrombus formation.^[30,31]^ This mechano-physiological mechanism may explain the paradoxically lower incidence of thromboembolic events in this population.

Similarly, a concave-shaped chest wall (defined by a manubrium-to-hip index [MHI] > 2.5) has been linked to reduced mid- to long-term cardiovascular risk. Sonaglioni et al propose that this anatomical configuration may optimize hemodynamic efficiency and enhance exercise tolerance, thereby lowering susceptibility to adverse cardiovascular events compared to individuals with normal chest morphology (MHI ≤ 2.5). These findings highlight the potential interplay between thoracic biomechanics, hemodynamic adaptation, and cardiovascular resilience, warranting further exploration in risk stratification models.^[32]^

Emerging evidence underscores the imperative for future cardiovascular prevention guidelines to adopt personalized strategies that integrate not only dietary and lifestyle modifications but also demographic and anthropometric variables. The relationship between age, sex, and stroke risk is dynamic, evolving across the lifespan. Younger women exhibit comparable or elevated stroke risk relative to men, largely attributable to pregnancy-related complications, oral contraceptive use, and hormonal fluctuations. In contrast, men demonstrate a marginally higher relative stroke risk in older age; however, the absolute burden of stroke remains greater in women due to their longer life expectancy.^[4]^ A multinational European cohort study further revealed a 9% annual increase in stroke risk among men and 10% among women, highlighting the cumulative impact of aging as a critical risk determinant.^[33]^ These findings advocate for age- and sex-stratified preventive protocols to enhance protection against CVD.

Future research priorities should include rigorous evaluation of community-based interventions aimed at mitigating disparities in cardiovascular outcomes, particularly in underserved populations. Concurrently, studies must assess the efficacy of targeted education initiatives in improving adherence to evidence-based lifestyle changes, such as physical activity, smoking cessation, and dietary optimization. By harmonizing individualized risk profiling with population-level strategies, healthcare systems can advance equitable, precision-driven approaches to CVD prevention.

## 5. Strengths and limitations

While previous observational studies have explored the relationship between dietary habits and stroke risk, this study employs an MR approach, utilizing genetic data to provide stronger causal inferences by minimizing confounding and reverse causation. By analyzing data from over 600,000 participants across 2 unique populations—UKB and FinnGen—the study offers a robust evidence of the significant role of fruit and salad consumption in reducing stroke risk. This genetic perspective strengthens the existing literature and adds a novel dimension to the evidence base. These findings not only inform public health dietary guidelines but also support targeted interventions and awareness campaigns aimed at promoting healthier eating habits, particularly in at-risk populations.

While our study provides valuable insights into genetic variants associated with stroke risk, cautious interpretation is warranted due to several limitations. One key limitation of this study is that the study’s exclusive focus on European ancestry populations limits the generalizability of our findings to other ethnicities.^[34]^ As the genetic data were derived from the UKB and FinnGen cohorts, this restriction limits the generalizability of the findings to other ethnic groups, such as Asian, African, and Hispanic populations, where genetic architecture, dietary habits, and environmental influences may differ significantly. Additionally, the potential influence of unaddressed confounders on the observed relationships cannot be ruled out. The presence of selection bias within the UKB, both at baseline and in terms of ongoing participation, is acknowledged in existing literature, emphasizing the need for careful consideration, and avoiding overly broad interpretations.^[35,36]^ UKB participants are known to be healthier and more socioeconomically advantaged than the general population. Future studies should validate findings in population-representative cohorts.

Another limitation of this study is its primary focus on food and lifestyle variables, neglecting demographic, anthropometric, and echocardiographic data, which are equally significant drivers of stroke risk. Variables like age, sex, body composition (BMI, waist circumference), cardiac architecture (e.g., left ventricular mass, mitral valve performance), and vascular health indicators may yield a more thorough comprehension of stroke vulnerability.

To overcome these limitations, replication studies in independent cohorts, especially those with diverse populations, are essential to validate our findings on a broader scale. Furthermore, delving into the underlying pathophysiology of the identified risk factors would offer valuable mechanistic understanding. Future research should also be expanded on MR analyses to untested dietary/lifestyle factors (e.g., specific nutrients, exercise) and explore mediating mechanisms (e.g., biomarkers) and population-specific effects. Updated guidelines should prioritize evidence-based dietary interventions (e.g., promoting fruit/salad intake) and integrate tailored public health strategies, emphasizing causal links to strengthen preventive messaging. Multidisciplinary studies assessing interactions between genetic, environmental, and behavioral factors could refine risk stratification and intervention efficacy. By addressing these limitations and expanding on our existing knowledge, future research has the potential to markedly enhance our understanding and management of stroke.

## 6. Conclusion

This two-sample MR study offers strong evidence in favor of possible causal relationships between dietary practices, lifestyle factors, and cardiovascular risk factors and the risk of stroke. These findings underline the impact of lifestyle choices and food patterns in determining stroke risk. In contrast to previous observational studies, this study utilizes MR to provide stronger causal evidence linking fruit and salad consumption to a reduced risk of stroke. These insights have important public health implications, reinforcing the need for dietary interventions and targeted awareness campaigns to promote healthy eating habits, particularly among populations at higher risk for stroke. Given the robustness of findings, we believe this study makes a meaningful contribution to cardiovascular prevention strategies. To fully understand the roles that additional dietary and lifestyle factors play in preventing strokes, more research on these topics should be conducted in the future.

## Author contributions

**Conceptualization:** Mohammad A. Jareebi.

**Data curation:** Mohammad A. Jareebi.

**Formal analysis:** Mohammad A. Jareebi.

**Funding acquisition:** Mohammad A. Jareebi.

**Investigation:** Mohammad A. Jareebi.

**Methodology:** Mohammad A. Jareebi.

**Project administration:** Mohammad A. Jareebi.

**Resources:** Mohammad A. Jareebi.

**Software:** Mohammad A. Jareebi.

**Supervision:** Mohammad A. Jareebi.

**Writing – original draft:** Mohammad A. Jareebi.

**Writing – review & editing:** Mohammad A. Jareebi.
